# Foam Splint versus Spica Cast—Early Mobilization after Hip Reconstructive Surgery in Children—Preliminary Data from a Prospective Randomized Clinical Trial

**DOI:** 10.3390/children9020288

**Published:** 2022-02-18

**Authors:** Lorenz Pisecky, Gerhard Großbötzl, Manuel Gahleitner, Christian Stadler, Stella Stevoska, Christina Haas, Tobias Gotterbarm, Matthias Christoph Michael Klotz

**Affiliations:** 1 Department for Orthopaedics and Traumatology, Johannes Kepler University Linz, Kepler University Hospital GmbH, Altenberger Strasse 96, 4040 Linz and Krankenhausstraße 9, 4020 Linz, Austria; gerhard.grossboetzl@kepleruniklinikum.at (G.G.); manuel.gahleitner@kepleruniklinikum.at (M.G.); christian.stadler@kepleruniklinikum.at (C.S.); stella.stevoska@kepleruniklinikum.at (S.S.); christina.haas@kepleruniklinikum.at (C.H.); tobias.gotterbarm@kepleruniklinikum.at (T.G.); 2 Department for Orthopaedics and Traumatology, Marienkrankenhaus Soest GmbH, Widumgasse 5, 59494 Soest, Germany; mcmklotz@gmx.net

**Keywords:** dysplasia of the hip, hip reconstructive surgery, spica cast, foam splint, complications, quality of life, prospective randomized clinical trial

## Abstract

Background: Surgical hip joint reconstruction may be the method of choice for children and adolescents with developmental dysplasia of the hip (DDH), as well as neurogenic dislocation of the hip (NDH) and Legg–Calvé–Perthes disease (LCPD). Following pelvic surgery, immobilization using a spica cast is considered to be the gold standard, despite the fact that casting may cause complications, such as hygienic problems, skin lesions, neurological deficits, and rigidity of the adjacent joints. An alternative for postoperative immobilization is a foam splint. The purpose of this randomized controlled trial was to compare spica cast and foam splint immobilization after hip reconstruction in children and adolescents with DDH, NDH, and LCPD. Methods: In a prospective randomized clinical trial, children and adolescents (age: 4–14 years), who received hip reconstructive surgery (osteotomy of the ilium and proximal femur, open reduction, soft tissue techniques) for DDH, NDH, and LCPD were included. Patient recruitment, group allocation, surgery, and aftercare were carried out in a department for orthopaedic surgery in Central Europe. Standardized questionnaires SF-36 (Short Form-36), EQ-5D (Euro Quality of Life 5D and CPCHILD (Caregiver Priorities and Child Health Index of Life with Disabilities) were gathered before, six, and twelve weeks after surgery from each patient. Group one received a spica cast and group two a foam splint for a period of six weeks postoperatively. There was no difference in surgical treatment. Results: Twenty-one out of thirty planned patients were enrolled in the study. One patient had to be excluded because of a lack of compliance. All quality of life (QOL) scores showed a significant reduction at the 6-week follow-up compared to the preoperative assessment. After twelve weeks, the scores came back close to the preoperative values. A significant reduction was seen in the spica cast group pre- vs. postoperatively for the variables CPCHILD (81% vs. 64%, *p* = 0.001), EQ-5d (65% vs. 45%, *p* = 0.014), and SF-36 (85% vs. 74%, *p* = 0.004). The corresponding values for the foam splint group also presented a reduction for all scores, but without statistical significance. Complications occurred in five cases. Conclusions: Recent retrospective studies suggest that foam splint immobilization after hip reconstruction surgery is a safe and feasible method, promising fewer complications compared to spica casting. The preliminary results of this prospective randomized clinical trial show an improvement of the scores when using a foam splint compared to the conventionally used spica cast. Benefits for the patients may be fewer adverse events and no need to undergo a second round of anaesthesia for recasting. Data suggest higher patient and caretaker satisfaction in the foam splint group.

## 1. Introduction

Developmental dysplasia of the hip joint (DDH) and deformations of the proximal femur may occur congenitally. Other diseases, such as cerebral palsy (CP) or myelomeningocele (MMC), can result in neuromuscular dysplasia of the hip (NDH) and/or dislocation of the hip [1]. Consequences may be pain when walking/standing/sitting, as well as difficulty in ambulating or even the inability to stand.

In early childhood, DDH or dislocation of the hip may be treated by casting or splinting. In case of failed conservative treatment, surgical reposition and reconstruction of the hip is needed.

Children with neuromuscular disorders show a high incidence of hip dislocations and failed conservative treatment due to NDH [2]. In CP, different authors found that hip dislocation occurred in 18–60% of their patients [3].

Besides DDH and NDH, young patients with Legg–Calvé–Perthes disease (LCPD) benefit from pelvic reconstruction, with the aim of improving the hip containment to prevent further lateralization or to support the rebuilding of the femoral head [4,5,6].

Especially in older ambulating children, tight soft tissue makes the procedure challenging. A combination of soft tissue and bony procedures may be necessary to achieve a reduction of the joint [7,8].

Postoperative immobilization is usually performed by spica casting for six weeks, followed by physiotherapy [9]. Most surgeons prefer casting to avoid secondary dislocation, especially in patients with spasticity.

A broad variety of complications is known: hygienic problems, skin lesions, neurological complications, and rigidity of the joints after casting. In some institutions, a change of the spica cast is performed in a second short round of general anaesthesia two weeks after surgery.

An alternative for postoperative immobilization is a foam splint. It protects the surgically treated hip from dislocation, provides access to the wound, and allows physiotherapy of the adjacent joints.

Retrospective studies showed the safety of the foam splint concerning the healing of the bone and promised fewer complications. Gather et al. showed in 2018 that foam splinting is not inferior to spica casting with regard to major complications such as the dislocation of the bone wedge, avascular necrosis of the acetabulum or femur, events of osseous non-union, or nerve injury [10].

Despite this retrospective data, there is no final consensus on the best means of postoperative immobilization in the treatment of DDH. It is assumed that foam splinting leads to a smaller number of complications and higher patient and caregiver satisfaction.

In a prospective randomized clinical trial, the study group now aims to test the hypothesis that the foam splint leads to a higher satisfaction of the patient and the caretaker by measuring well-established quality of life (QOL) scores pre- and postoperatively.

Furthermore, a lower number of complications is expected. Benefits for the patients may be fewer unplanned outpatient contacts, a higher quality of life during the aftercare process, and no need to undergo a second round of anaesthesia for recasting. Up to now, a comparable study does not exist, as trials dealing with foam splinting after pelvic reconstruction were performed in most cases retrospectively and/or were non-randomized [10,11,12].

## 2. Materials and Methods

The study group of a Central European department for orthopaedic surgery designed a non-blinded, prospective randomized clinical trial to test the following hypothesis: foam splinting leads to a higher postoperative satisfaction of patients and their caretakers in cases of pelvic reconstruction.

Ethical approvement was obtained prior to the conduction of the clinical trial (Ethikkommission des Landes Oberösterreich EK Nr: 1183/2018), and a scientific grant by the Medical Society of Upper Austria funds the research. The clinical trial was registered at the German Clinical Trials Register after ethical approval (DRKS-ID: DRKS00016861).

Patients were recruited in the outpatient clinic for paediatric orthopaedics and neuro-orthopaedics at the study site. Recruitment was performed by a single paediatric orthopaedic surgeon with more than 20 years of clinical experience.

Included patients were children and adolescents from 4 to 14 years of age with the diagnoses ‘congenital dysplasia of the hip’, ‘neuromuscular dysplasia of the hip’, and ‘Legg–Calvé–Perthes disease’, with indication for hip reconstructive surgery. Indications for surgery were a Reimers migration index of 40% or higher or 25–40% with progression, Tönnis classification II or higher, or an AC-Index above the Tönnis standard and head-at-risk signs in cases of LCPD.

**Figure 1:**
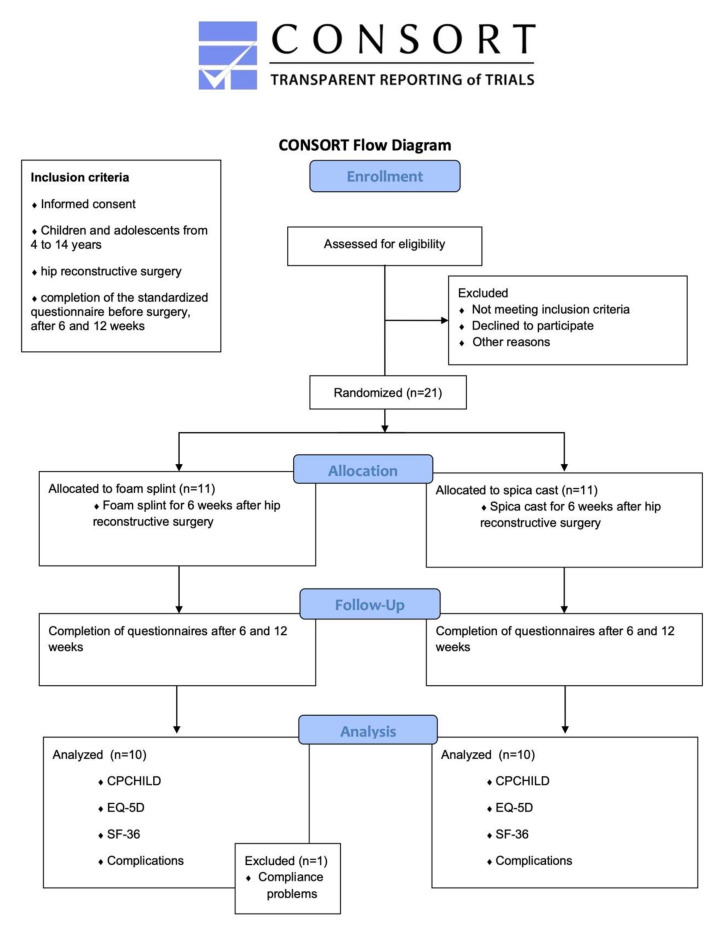
The algorithm of the trial; CONSORT diagram adapted from [13].

The standardized questionnaires (SF-36, EQ-5D, and CPCHILD) were completed before surgery and after 6 and 12 weeks, and participation was mandatory. Post-trial care was performed within a standardized yearly routine check-up and did not differ in the two groups. All planned postoperative follow-up checks were conducted at the outpatient clinic for paediatric orthopaedics and neuro-orthopaedics at the study site by one senior and one junior paediatric orthopaedic surgeon. The algorithm of the recruitment process is shown in Figure 1.

Obtained informed consent from the patient and legal guardian was mandatory and collected by the surgeon prior to the procedure.

Two groups with differing immobilization protocols were formed.

**Figure 2:**
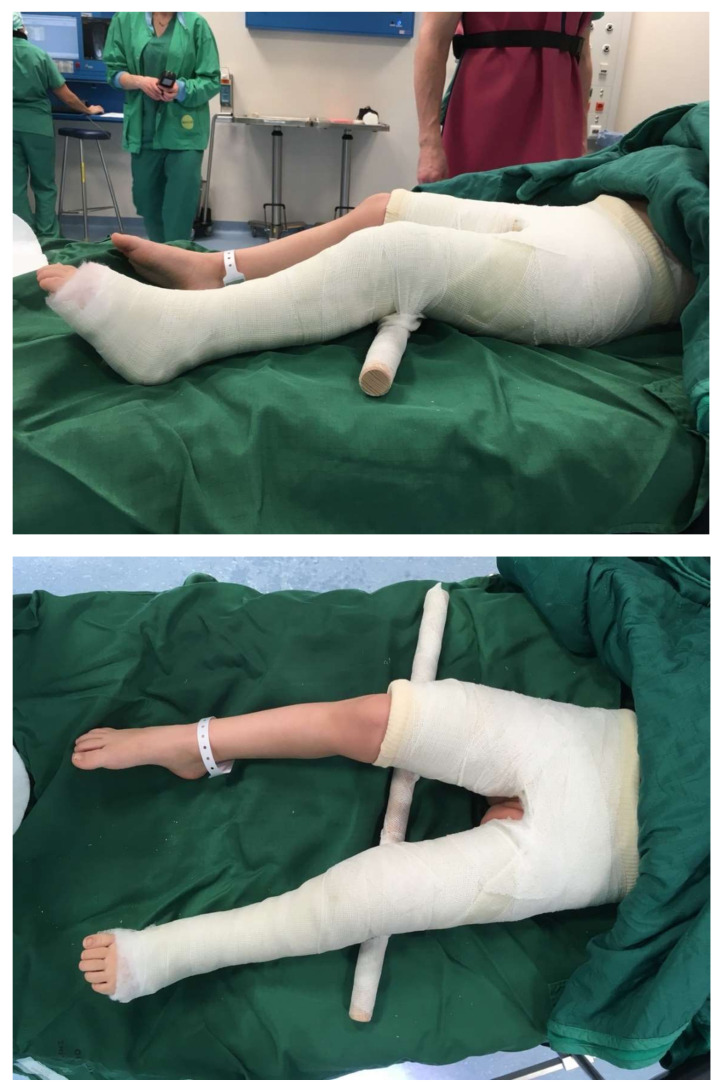
Postoperative spica cast.

Group one, the control group, was treated using a standardized spica cast with slight flexion of the hip of about 10–15 degrees, 10 degrees inward rotation of the hip and 30 degrees of abduction of the hip (Figure 2). Two weeks after surgery, a second short general round of anaesthesia for the removal of the skin suture and to change the cast was necessary.

**Figure 3:**
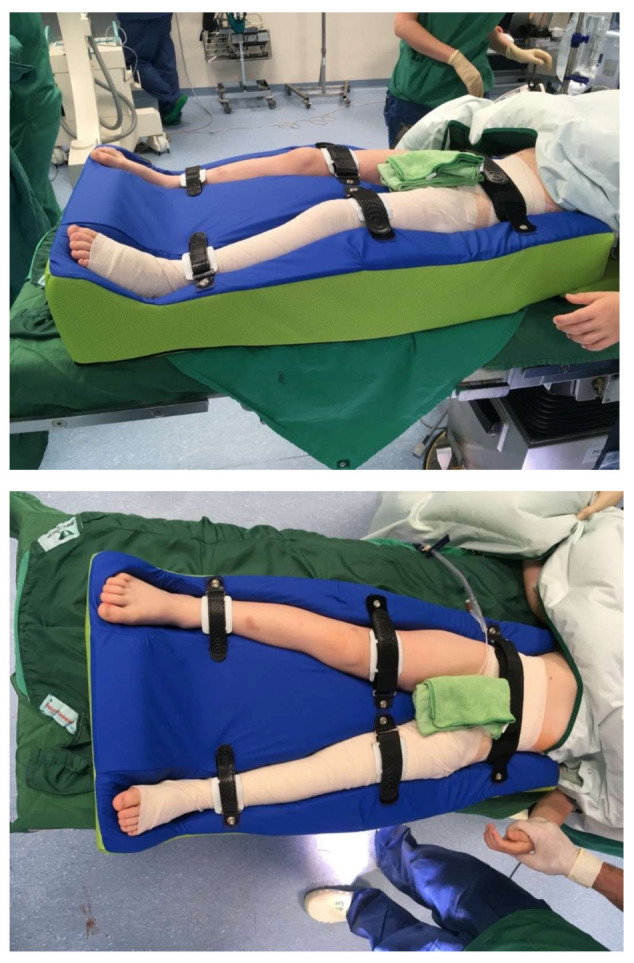
Postoperative foam splint.

Group two, the intervention group, was treated using immobilization with a foam splint for six weeks with slight flexion of the hip of about 10–15 degrees, 10 degrees inward rotation of the hip and 30 degrees of abduction of the hip (Figure 3). No second round of anaesthesia for recasting in the operating room was needed in group two. During the period of immobilization in group two, physiotherapy of the lower extremity was performed considering the patient’s needs by experienced neuro-paediatric physiotherapists.

The assignment process to the two groups was random, using a coin toss four to six weeks prior to surgery. The randomization process was performed by a junior paediatric orthopaedic surgeon, and observed by a senior paediatric orthopaedic surgeon in awareness of the possibility of unintentional unbalanced results [14]. The randomization process was chosen as the simplest method and to avoid manipulation by the change of chronological order, as would be possible in a fixed randomization sequence.

There was no difference in the surgical technique between both groups. Included surgical techniques were derotating varisation osteotomy of the femur (DVO), Pemberton acetabuloplasty, and Salter and Chiari osteotomy. Soft tissue techniques were tenotomy of the psoas muscle, of the adductor muscles, of the knee flexors, and the lengthening of quadriceps tendon.

Standardized questionnaires were used to measure the quality of life and the quality of aftertreatment 6 and 12 weeks after surgery (Table 1). The questionnaires used were the ‘Caregiver Priorities and Child Health Index of Life with Disabilities’ (CPCHILD), the ‘Short Form 36’ (SF-36), and the ‘Euro Quality of Life 5D’ (EQ-5D) [15,16,17]. The evaluation of the questionnaires was performed by a junior paediatric orthopaedic surgeon. Mobility was measured using the ‘Gross Motorfunction Classification System’ (GMFCS) scale [18].

The main hypothesis to test was: foam splinting for immobilization after hip reconstructive surgery leads to a higher satisfaction of the patients and a higher quality of care than spica casting, measured with the parameter ‘Caregiver Priorities and Child Health Index of Life with Disabilities’ (CPCHILD) [15,19].

The side issue was: are there fewer complications, such as hygienic problems, skin lesions, and neurological complications?

The clinical endpoint of the study was the completion of the 12-week postoperative questionnaire. Only patients with full completion of the questionnaires were included in the further statistical analysis. Criteria for exclusion were a lack of consent and cooperation.

Data storage and analysis were performed pseudonymized, and only study authors have access to the full datasets.

Statistical methods included a detailed epidemiological description with mean and standard deviation, minimum, maximum, and median for continuous data and scores, and relative frequency for all variables. The main characteristics were analysed with a *t*-test or chi-square test. Values for *p* are given and values <0.05 are considered to be statistically significant. When appropriate, graphical methods for visualisation are used.

The calculation of sample size used a two-sided *t*-test (difference between two independent means), effect of Cohen’s d 1.1, alpha = 0.05, 1-beta = 0.80, and allocation ratio 1:1, and this resulted in a number of 15 patients per group (Software G*Power 3.1). Statistical analysis was planned in accordance with the local institute for biomedical statistics.

### Patient and Public Involvement

The planning study was supported by the local ethical review committee, including the representative for disabled patients, which provides professional ethical and legal advice. The ethical review committee reviewed the study protocol during the planning of the clinical trial and gave advice on ethical and legal topics, as well as statistical issues. The committee partnered with the study group for the design of the study, the informational material to support the intervention, and the burden of the intervention from the patient’s perspective.

## 3. Results

Twenty-one out of thirty planned patients were involved (Table 2) until the end of 2021. One patient had to be excluded from further analysis due to parental compliance problems using the foam splint. This patient had to be casted two weeks postoperatively and further postoperative regime was unremarkable.

A complete dataset is available for 20 patients, wherein 10 received a spica cast and 10 a foam splint.

Of 15 patients with NDH, 0 were GMFCS type I, 1 was type II, 3 were type III, 2 were type IV, and 9 were type V. Eight of these patients received a spica cast, showing the efficiency of the randomization process.

Complications occurred in five patients. In the foam splint group, two patients suffered from superficial skin lesions on their medial malleolus. Both cases were seen in children with spasticity of the lower extremity. Analysing those cases revealed that the foam splint was too tight around the ankles. The design of the foam splint had to be adapted for the following cases by the orthopaedic technician. The superficial skin lesions were draped with foam patches and healed within one week. One patient of the foam splint group suffered from a deep wound infection of the proximal lateral femur 4 days postoperatively. The wound had to be revised surgically on the fifth postoperative day and healed under antibiotic treatment within two weeks.

In the spica cast group, one patient showed a superficial skin lesion on the heel caused by the cast. This was seen two weeks postoperatively during the planned recasting. The second cast ended just above the ankle, the heel was covered with foam patches and healed within two weeks. One patient of the spica cast group suffered from a deep wound infection of the groin five days postoperatively and had to be revised surgically on the sixth day postoperatively. The wound healed under antibiotic treatment within two weeks. In none of the patients did the osteosynthesis material have to be revised.

The quality of life (QOL) scores showed a significant reduction at the 6-week follow-up compared to the preoperative assessment. The scores came back close to the preoperative values until the second postoperative assessment after twelve weeks (Table 3).

A significant reduction of all QOL scores was seen in the spica cast group pre- vs. postoperatively for the variables CPCHILD (81% vs. 64%, *p* = 0.001), EQ-5d (65% vs. 45%, *p* = 0.014), and SF-36 (85% vs. 74%, *p* = 0.004).

Furthermore, the values for the foam splint group also presented a reduction but did not show statistical significance. (Table 4).

**Figure 4:**
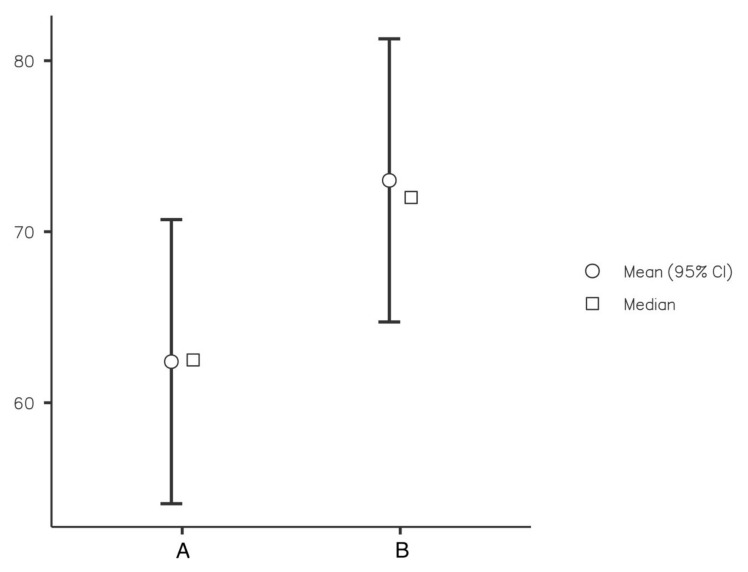
CPCHILD score at the 6-week follow-up for spica cast (A) and foam splint (B).

Six weeks after surgery, values for CPCHILD (Figure 4), EQ-5D, and SF-36 were lower in the spica cast group than in the foam splint group, despite the fact that the difference did not reach the level of statistical significance (CPCHILD 73% vs. 64%, *p* = 0.147). The difference between the two groups decreased until the 12-week follow-up, still without statistical significance.

## 4. Discussion

Clinical trials on children and handicapped persons need to be carried out under sound ethical and scientific standards, considering their personal and legal independency.

Participation in a clinical trial needs in-depth discussion with the legal guardian and appropriate discussion with the child, depending on age and capacity.

Dysplasia of the hip must be treated prior to the ability to give informed consent to prevent deterioration or even loss of gait and posture. Therefore, it must be clarified to the patient and the guardian that hip reconstructive surgery is not only a measure to improve function, but also the quality of life for many years.

There is evidence that hip reconstructive surgery has a positive effect on the quality of life of children with cerebral palsy: DiFazio et al. were able to show the positive effect of hip reconstructive surgery in children with cerebral palsy in 2016 [15].

Despite the decades-long history of surgical correction of the hip and pelvis, there is no final consensus about the ideal modality or duration of postoperative immobilization, although the discussion is vivid and controversial [9].

Complications associated with spica cast immobilization are known, and recent publications have shown adverse events in 4.5% to 13.4% of cases in hip reconstructive surgery and up to 28of in cases in femoral fractures treated with spica casts [11,20]. The study group of Pisecky et al. presented a complication rate of 27.3% using spica cast immobilization in hip reconstructive surgery. Complications were more common in patients with NDH (35%) than DDH (22%). Nine out of twenty-three adverse events were considered to be cast-associated [21].

Aiming for the reduction of superficial skin lesions, Murgai et al. presented data for foam padding of the spica cast in 920 patients with 2481 immobilizations. It was possible to show a decrease in the complication rate in A-frame casts from 13.4% to 4.5%. Patients with neurologic disorders had the lowest complication rate with 0.7%, and neurovascular deficits were described in none of the cases with foam padding, in contrast to 4.5% in patients without additional measures [11].

DiFazio et al. presented a complication rate of 28% in cases treated with spica cast immobilization for femoral fractures. Thirty-one percent of the patients needed a readmission for recasting [20]. In a prospective trial, the same group was able to show a reduction of the complication rate from 13.6 to 6.6 cases per 1000 castings by using foam pads to enhance the cast [12].

As a study group with a large amount of experience in the usage of foam splints, Gather, Dreher et al. established a straightforward immobilization protocol with early physiotherapeutical remobilization and full weight bearing four weeks postoperatively after immobilization. According to the results of the study, no device-associated complication was seen. Therefore, it can be assumed that foam splinting is as safe as spica casting concerning the postoperative immobilization for the fixation of the osseous result [10].

Nevertheless, these reports are retrospective case series, and high-quality prospective randomized clinical trials are lacking. A broad variety of feasible aftercare procedures is known and, in most institutions, the surgeon’s preference, personal experiences, or just tradition is the reason given for a type of postoperative immobilization. It is thought to be a compromise to protect the result of the surgical procedure on one hand and to prevent superficial skin lesions or worse complications on the other. The fear from loss of correction stands in contrast to a short and complication-free period of immobilization, as desired by the patient and the caretaker. Mostly, minor adverse events, such as skin lesions, superficial wound healing problems, and hygienic challenges, are responsible for many unscheduled outpatient contacts. These unplanned contacts bind medical professionals to time-consuming procedures and lead to rising costs in the health care system. More severe complications, such as deeper lesions of the skin, may need unplanned readmission to hospital, and in some cases surgical revision, with the risk of further adverse events. Interfering with the patient’s autonomy and the caretaker’s independence, readmissions and surgical procedures should be avoided by averting the underlying complication. Apart from the patient-related drawbacks, the financial burden of unplanned readmissions to hospital and recasting is enormous. In 2011, DiFazio calculated expenses of USD 12,719 for readmission and recasting in the operating room under sedation [20]. Furthermore, unplanned procedures take up necessary time in the operating room and, in the worst case, postpone planned surgical procedures.

In order to improve evidence relating to postoperative treatment, this is the first prospective randomized clinical trial comparing spica casts and foam splints for postoperative immobilization in patients who received hip reconstructive surgery. The purpose of this ongoing study is to evaluate quality of life and complication rates between these two different immobilization devices, and the preliminary data show promising results.

Until today, spica cast immobilization is still a widely used regimen of aftertreatment following pelvic surgery in children. It is necessary to provide criteria for the usage of a foam splints instead of casting in immobilization after hip reconstructive surgery.

### Strengths and Limitations

Providing the first prospective randomized clinical trial on the topic of foam splinting and casting in immobilization after hip reconstructive surgery, the obtained data may support decision making in postoperative care concerning the safety and satisfaction of patients and caretakers. This clinical trial was planned under high ethical and legal standards and has passed the ethical review committees. At this point, the main limitation to achieve statistical significance may be the small number of participants of 10 patients in each group. The present data show the necessity to include at least 17 patients in each group to reach levels of statistical significance. More patients must be included in the study and further trials may be needed to obtain reliable data.

## 5. Conclusions

The data suggest higher patient and caretaker satisfaction in the foam splint group. The preliminary results of this clinical trial show an improvement of the QOL scores when using a foam splint compared to the conventionally used spica cast. Nevertheless, the values do not show statistical significance, most likely because of the low number of patients included and the relatively small difference. The conduction of the clinical trial is necessary.

## Figures and Tables

**Table 1:** Schedule of enrolment, interventions, and assessments.

	STUDY PERIOD				
	Enrolment	Allocation	Post-Allocation		Close-Out
TIMEPOINT	−t1	0	t1	t2	t3
ENROLMENT:	X				
Eligibility screen	X				
Informed consent	X				
Allocation		X			
INTERVENTIONS:					
Spica Cast			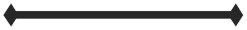		
Foam Splint			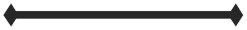		
ASSESSMENTS:		X	X	X	X
CPCHILD		X		X	X
SF-36		X		X	X
EQ-5D		X		X	X
Complications screen			X	X	X

t1 = surgery, t2 = 6 weeks after surgery, t3 = 12 weeks after surgery. X = action; 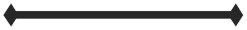 = interventional process.

**Table 2:** Epidemiologic data and procedures.

	DDH	NDH	LCPD
N (hips)	4	15	2
age at surgery	5.4y ± 3.6	8.0y ± 3.0	6.5y ± 0.6
m:f	2:2	8:7	2:0
right:left	2:2	8:7	0:2
bilateral	0	0	0
Surgical procedure in detail			
Femoral osteotomy	4	15	2
Osteotomy of ilium	4	15	2
Salter osteotomy	0	0	2
Chiari osteotomy	0	1	0
Pemberton osteotomy	4	14	0
Psoas tenotomy	0	3	0
Adductor tenotomy	0	4	0
Open reduction	2	6	0
Hamstring lengthening	0	2	0
Lengthening of extension mechanism	0	1	0

**Table 3:** Comparison of QOL scores at the three assessments.

	CPpre	Low–High	SD	EQ-5Dpre	Low–High	SD	SF-36pre	Low–High	SD
Total	77%	50–100	20	62%	24–100	25	82%	63–99	10
Foam splint	74%	50–100	22	60%	24–100	26	80%	63–99	11
Spica cast	81%	52–100	16	65%	37–94	24	85%	72–97	9
diff	7%		10	5%		12	5%		5
*p*	0.595			0.803			0.360		
	CPpost1	low–high	SD	EQ-5Dpost1	low–high	SD	SF-36post1	low–high	SD
Total	69%	36–89	14	49%	35–81	13	76%	66–87	5
Foam splint	73%	58–89	13	53%	38–81	15	78%	73–87	5
Spica cast	64%	36–78	14	45%	35–68	10	74%	66–80	5
diff	9%		7	8%		6	4%		2
*p*	0.147			0.253			0.087		
	CPpost2	low–high	SD	EQ-5Dpost2	low–high	SD	SF-36post2	low–high	SD
Total	69%	36–99	15	66%	34–98	19	84%	72–97	7
Foam splint	79%	60–99	15	65%	41–98	15	87%	78–97	5
Spica cast	74%	36–78	14	66%	34–68	17	82%	72–91	7
diff	5%		6	1%		9	5%		3
*p*	0.385			0.873			0.110		

**Table 4:** Comparison of pre- and postoperative QOL scores, split up by mode of immobilization.

	CP	Low–High	SD	EQ-5D	Low–High	SD	SF-36	Low–High	SD
Spica cast pre	81%	52–100	16	65%	37–94	24	85%	72–97	9
Spica cast post1	64%	36–78	14	45%	35–68	10	74%	66–80	5
diff	17%		4	20%		7	9%		4
*p*	0.001			0.014			0.004		
	CP	low–high	SD	EQ-5D	low–high	SD	SF-36	low–high	SD
Foam splint pre	74%	50–100	22	60%	24–100	26	80%	63–99	11
Foam splint post1	73%	58–89	13	53%	38–81	15	78%	73–87	5
diff	1%		4	7%		6	2%		2
*p*	0.965			0.269			0.437		

## Data Availability

The data presented in this study are available on request from the corresponding author. The data are not publicly available due to data protection policy involving children and patronized subjects.
